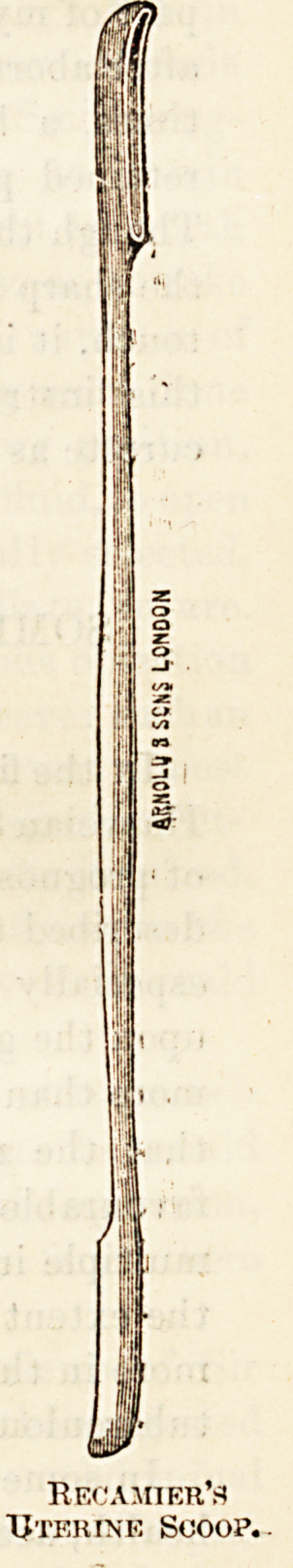# Treatment of Chronic Cervical Catarrh of the Uterus

**Published:** 1900-12-08

**Authors:** George A. Hawkins-Ambler

**Affiliations:** Surgeon to the Samaritan Hospital for Women, Assistant Surgeon, Stanley Hospital, Liverpool


### Hospital Clinics and Medical Progress.

### TREATMENT OF CHRONIC CERVICAL CATARRH OF THE UTERUS.

(*Continued from page* 152.)

By George A. Hawkins-Ambler, F.R.C.S.E., Surgeon to the Samaritan Hospital for Women, Assistant Surgeon, Stanley Hospital, Liverpool.

Place the patient in the lithotomy position; draw her to the edge of the couch or table when anæsthetised, insert the speculum; after shaving and disinfecting the genitals, swab the vagina thoroughly with lotion. You may also take the opportunity of thoroughly examining the pelvis to obtain a clear idea of the condition of the ovaries and Fallopian tubes. Now seize the anterior lip of the cervix with a volsellum and draw it down, and insert one into the posterior lip also. In doing this, be careful not to pinch up the cervix transversely, so narrowing the canal for the dilators; and do not have the hooks of the volsellum projecting into the cervical canal to scratch the dilators and confuse you. Next pass a sound to discover the depth, size and position of the uterine cavity. The nurse or assistant will now pass you the dilators in their numerical order, first dipping them in the lubricant. Insert them gently into the cervical canal in the direction of the uterine cavity. If you have not previously ascertained this by means of the uterine sound, you may attempt to pass a dilator in a forward direction into a uterus that is retroflexed, thus driving the dilator through the uterine wall or experiencing troublesome resistance in introducing it. Press the instrument steadily, and if it have been warmed by insertion in hot lotion or sterilised water previously to being lubricated, it will pass in the uterine cavity. Some resistance will be experienced at the internal os, but this is soon overcome if the dilator is held with the broad end in the palm of the right hand, while the forefinger is placed about two inches from the point, to prevent a sudden plunge forward of the instrument when it is inserted into an unexpectedly relaxed cervix, while the left hand makes counter pressure by drawing the cervix against the dilator or fixing it firmly. If undue haste is used there is danger of tearing the cervix—an accident that is indicated by a tinge of blood, or hæmorrhage that may require a catgut suture to control it and minimise the risk of cellulitus. When the dilator is well within the uterus, slide it gently backwards and forwards once or twice till it runs easily, withdraw it, and at once introduce the next size. Proceed in this way until the uterus is sufficiently dilated to admit the easy passage of the Simon's spoon. This is usually at about No. 7 or 8 in my dilators, corresponding to No. 12 to 14 Hegar. The beginner is sometimes alarmed to find that, after passing two or three dilators, there is a large increase in the size, depth, and capacity of the uterus. This gives the impression that it has ruptured, the more so in uteri with thin and softened walls. It is not so, however; and the cautious introduction of the curette or sound, which may be passed gently over the uterine walls, will indicate no lesion. If the accident has happened, no harm will result if your antiseptic precautions have been carefully devised. Do not in this event proceed with the operation, but carefully pack a strip of gauze into the uterine cavity to the site of the tear or to the fundus, swab the vagina with cotton dipped in lotion, and return the patient to bed with an antiseptic pad over the genitals, leaving the gauze for 48 hours before withdrawing.

When the uterus is sufficiently dilated, douche the cavity with lotion and introduce the spoon. It is essential that you proceed systematically to scrape the uterine walls from top to bottom, beginning at a definite point and proceeding all round the cavity, leaving no strip of unscraped mucosa. Scrape the soft or rugged mucosa away till you feel the muscular tissue firm beneath the edge of the spoon. When this has been done, scrape the cervix still more thoroughly, removing the mucosa. Next, take the smaller curette (Récamier's) and scrape out the angles of the uterus which the broader spoon does not so readily attack. Now douche the interior of the uterus with lotion, using a double-channel catheter, or wipe it with dressed skewers damped with lotion. Whether the douche is used or not, thoroughly dry the cavity with the dressed skewers or probes. Now dip another dressed probe in phenol or iodised phenol and thoroughly swab the interior of the uterus, repeating the process if there be any doubt about the completeness of the proceeding. Wash away with lotion any excess that may have run into the groove of the speculum. This swabbing causes some contraction of the uterus, and before introducing the gauze drain, it will facilitate matters if you dip the end into the lubricant to enable it to pass the internal os more easily. The gauze is next passed into the uterus by means of the sound or a small dilator, not tightly, if there is no marked hæmorrhage, in order to drain the cavity. The gauze is left hanging out of the cervix in the vagina, another douche is applied for a moment, the volsellæ removed, the speculum withdrawn, and a pad of wool or a sanitary towel applied to the genitals. Replace your patient in bed, and keep her there for four to seven days. In 48 hours, after thoroughly cleansing and disinfecting your hands and cleansing the patient's genitals, you may withdraw the gauze drain, and give an antiseptic douche. Do this yourself; it should not be left to a nurse. I have known nurses and practitioners leave a part of the gauze behind by rough or careless withdrawal, and the patient die from pyo-salpinx. I always place the patient on a bedpan, or, better, draw her to the edge of the bed over a mackintosh sheet, withdraw the pack, and give the first douche myself. At this time, too, I order a saline purge, and when it has acted, place the patient on ordinary diet in place of the slops she lives on the first two days.

Récamier's Uterine Scoop.

I should say that some gynæcologists dilate slowly with laminaria tents before operation, or, as Lawson Tait practised, dilate with Tait's dilators before curetting. This avoids the risk of tearing the cervix; but I do not think much harm results from this accident if antisepsis is secured, and it is more trouble to doctor and patient. Tait also applied the actual cautery to the inner wall of the

B

uterus after curetting; but I have relinquished this in favour of the more penetrating phenol. We never remove all the glands in curettage, since these project into the muscular wall, and a thorough application of phenol is more effective than the cautery, the glands which are renewed in a few days, being subjected to the antiseptic action of the application. Where the uterine cavity is enlarged from metritis or endometritis, I find that the vigorous scraping of the cervix results in a decrease of this enlargement in many instances.

I should say, in conclusion, that one advantage of the use of the sharp spoon is that the uterine wall may be felt with the back of the spoon and localised with less risk of pushing it through the organ, as it is broader than a Récamier curette; and, though it scarcely comes into this part of my subject, it is as well to state that for curetting after abortion, where the tissues are soft and friable at times, a blunt wire curette is safer, as it removes the retained placenta and leaves less raw, absorbing surface. Though the skilled gynæcologist can do all he wants with the sharp spoon, owing to his experience and delicacy of touch, it is not wise for the general practitioner to adopt this instrument in all cases. I regard the flushing curette as an instrument of little value.

## Figures and Tables

**Figure f1:**